# Hospitalization and death among patients with influenza, Guatemala, 2008–2012

**DOI:** 10.1186/s12889-019-6781-6

**Published:** 2019-05-10

**Authors:** Trong Ao, John P. McCracken, Maria Rene Lopez, Chris Bernart, Rafael Chacon, Fabiola Moscoso, Antonio Paredes, Leticia Castillo, Eduardo Azziz-Baumgartner, Wences Arvelo, Kim A. Lindblade, Leonard F. Peruski, Joe P. Bryan

**Affiliations:** 1 Division of Global Health Protection, Center for Global Health, Centers for Disease Control and Prevention (CDC), 1600 Clifton Road NE, MS E-04, Atlanta, GA 30329 USA; 2 Centro de Estudios en Salud, Universidad del Valle de Guatemala, Guatemala City, Guatemala; 3 Ministry of Public Health and Social Welfare, Guatemala City, Guatemala; 4 Division of Influenza, National Center for Immunization and Respiratory Disease, CDC, Atlanta, USA; 5 Global Disease Detection Program, CDC Central America Regional Office, Guatemala City, Guatemala

**Keywords:** Influenza, Guatemala, Respiratory disease

## Abstract

**Background:**

Influenza is a major cause of respiratory illness resulting in 3–5 million severe cases and 291,243-645,832 deaths annually. Substantial health and financial burden may be averted by annual influenza vaccine application, especially for high risk groups.

**Methods:**

We used an active facility-based surveillance platform for acute respiratory diseases in three hospitals in Guatemala, Central America, to estimate the incidence of laboratory-confirmed hospitalized influenza cases and identify risk factors associated with severe disease (defined as admission to the intensive care unit (ICU) or death). We enrolled patients presenting with signs and symptoms of acute respiratory infection (ARI) and obtained naso- and oropharyngeal samples for real-time reverse transcriptase polymerase chain reaction (RT-PCR). We used multivariable logistic regression to identify risk factors for ICU admission or death, adjusted for age and sex.

**Results:**

From May 2008 to July 2012, among 6326 hospitalized ARI cases, 446 (7%) were positive for influenza: of those, 362 (81%) had influenza A and 84 (18%) had influenza B. Fifty nine percent of patients were aged ≤ 5 years, and 10% were aged ≥ 65 years. The median length of hospitalization was 5 days (interquartile range: 5). Eighty of 446 (18%) were admitted to the ICU and 28 (6%) died. Among the 28 deaths, 7% were aged ≤ 6 months, 39% 7–60 months, 21% 5–50 years, and 32% ≥ 50 years. Children aged ≤ 6 months comprised 19% of cases and 22% of ICU admissions. Women of child-bearing age comprised 6% of cases (2 admitted to ICU; 1 death). In multivariable analyses, Santa Rosa site (adjusted odds ratio [aOR] = 10, 95% confidence interval [CI] = 2–50), indigenous ethnicity (aOR = 4, 95% CI = 2–13, and radiologically-confirmed pneumonia (aOR = 5, 95% CI = 3–11) were independently associated with severe disease. Adjusted for hospital utilization rate, annual incidence of hospitalized laboratory-confirmed influenza was 24/100,000 overall, 93/100,000 for children aged < 5 years and 50/100,000 for those ≥ 65 years.

**Conclusions:**

Influenza is a major contributor of hospitalization and death due to respiratory diseases in Guatemala. Further application of proven influenza prevention and treatment strategies is warranted.

## Background

Influenza is a major cause of respiratory illness resulting in 3–5 million severe cases and 291,243–645,832 deaths annually [1–5]. Although comprising a small proportion of all influenza cases, patients requiring hospitalization for influenza-related illness suffer substantial health complications and financial burden to health systems. Recent advances in prevention with vaccines and antivirals can reduce morbidity and mortality from influenza illness, especially in those at high risk.

Despite its public health and economic importance, the incidence of influenza-related hospitalization has been estimated only in some locations [6–8], and there is insufficient quality data for Central America [9]. In El Salvador, the incidence of influenza-associated severe pneumonia was 3.2 cases per 1000 person years from 2008 to 2010 among children under five years of age [10]. In Guatemala, Lindblade, et al*,* described the clinical presentations of pandemic A(H1N1)pdm09 and seasonal influenza A (H1N1 and H3N2) from 2008 to 2009, but did not estimate the incidence for these illnesses [11]. They concluded that each caused substantial disease in infants. A prospective study in Nicaragua confirmed higher rates in infants aged 6–11 months than in those 0–5 months [12].

In addition, although common risk factors for illness attributed to influenza requiring hospitalization have been established, little is known about risk factors associated with poor outcomes among hospitalized cases, especially in Central America [13]. For instance, HIV infection is significantly associated with hospitalization for influenza in some parts of the world [14–16]. Other risks associated with hospitalization for influenza include pre-existing cardiac or neurologic and neuromuscular disease among children [7] especially those aged  < 2 years. Among adults, increased risk of complications include obesity, especially body mass index (BMI) > 40; age > 65 years, chronic medical problems such as diabetes and asthma, and pregnancy and infection during the two weeks post-partum [17, 18]. Symptoms and signs such as dyspnea, and pleural effusion, lymphopenia, and lack of or delayed antiviral treatment [19–21] have been associated with hospitalization and/or death. In some studies, certain ethnic groups suffered increased rates of hospitalization and death [17, 21]. For A(H1N1)pdm09 strain, being younger than 25 years was a reported risk factor for hospitalization [21]. Since the epidemiologic profiles are disparate globally, these conclusions may not be generalizable.

In 2004, the Technical Advisory Group (TAG) of the Pan American Health Organizations (PAHO) recommended vaccination of older adults (with priority for adults aged > 60 years), those with chronic illness, immune deficiencies, health professionals, pregnant women, and children 6–23 months [18]. Guatemala began to follow this recommendation in 2007. Few countries in the Americas, including Guatemala, have achieved high coverage among groups targetted for vaccination [22].

In Guatemala, influenza continues to be an important cause of illness [9]. Information about the most severe cases in the country might be useful to assess the value of influenza prevention and control programs in tropical middle-income countries like Guatemala. Our objective is to estimate the incidence of influenza hospitalization and in-hospital deaths from PCR-proven influenza in a stable, facility-based, active surveillance platform among ethnically and geographically diverse populations, and to identify risk factors associated with intensive care unit admission or death.

## Methods

### Facility-based surveillance system

Beginning in 2007, the Centers for Disease Control and Prevention of the United States (CDC), the Ministry of Public Health and Social Assistance [*Ministerio de Salud Publico y Asistencia Social* (MSPAS)] of Guatemala, and the *Universidad del Valle de Guatemala* (UVG) implemented an active facility-based surveillance system in the department of Santa Rosa supported by dedicated nurses and lab personnel whose main objective is to monitor diarrheal, respiratory, neurological, and acute febrile syndromes at the three main levels of health facilities (health post, health center, and hospital). This facility-based surveillance system is known as *Vigilancia Integrada Comunitaria* (VICo) or Integrated Community Surveillance.

Santa Rosa department has a population of approximatelyt 346,000, 3% of whom are of indigenous ethnic origin, according to the 2002 Census. The public hospital is located in Cuilapa at an elevation of 932 M (3057 ft) with a tropical climate and is the only public hospital in the department. In 2009, the surveillance system was replicated in the department of Quetzaltenango, which has a population of 789,000 that is 54% indigenous Mayan. The National Hospital of Quetzaltenango is located at 2407 M (7880 ft) (sub-tropical climate) and serves the highland region of western Guatemala. Additionally, respiratory surveillance was also conducted in Guatemala City from 2009 to 2011 in response to the influenza pandemic at the *Instituto Guatemalteco Seguridad Social* (IGSS), which has an elevation of 1512 M (4960 ft). Patients and their employers pay into the IGSS system while public hospital access is without charge.

Patients who presented at one of these hospitals (Cuilapa Regional Hospital, National Hospital of Quetzaltenango, and IGSS) with any symptoms of respiratory disease were invited to undergo screening. Patients who met enrollment criteria (see below) and provided written informed consent were asked to provide nasopharyngeal and oropharyngeal swabs and urine samples. These were stored at -20 °C and transported to a central laboratory at UVG under dry ice. Trained staff administered a face-to-face interview to collect patient demographic, clinical, and risk factor information using a personal digital assistant (PDA). Chest radiographs (CXRs) were performed as part of routine clinical care; surveillance staff obtained a digital image of CXRs done on enrolled patients using a digital camera [23–25]. The digital images were reviewed by a panel of radiologists who had undergone training on the World Health Organization (WHO) guidelines for standardized interpretation of CXRs for the diagnosis of pneumonia in children [25]. A modified version of the guidelines was used to interpret CXRs of adults, which included recording the same radiologic endpoints as are used for pediatric CXRs [26]. All digital images were reviewed independently by two radiologists and were classified as having consolidation or obscuring pleural effusion as an end-point, other consolidation/infiltrate, no consolidation/infiltrate/effusion or uninterpretable. In cases of discordant interpretations between the first two readers, a third interpretation was recorded and the final classification was determined by the majority. Consolidation was considered suggestive of a bacterial etiology [25].

### Surveillance case definitions [27]

If a patient was five years or older, the case definition for acute respiratory infection (ARI) was:

- At least one of the following: documented fever (≥38^o^C) in the past 7 days; temperature <35.5^o^C with chills; or abnormal white blood cell count (>11,000/mm^3^ or <3,000/mm^3^) or abnormal white blood cell differential AND
- At least one of the following: tachypnea, cough, sputum production, hemoptysis, chest pain, dyspnea, shortness of breath, sore throat, or abnormal lung exam.

If a patient was less than five years old, the Integrated Management of Childhood Illness (IMCI) guideline criteria [28] for management of pneumonia-like illness was applied in 2011:

1. Age <2 months with tachypnea or chest in-drawing, OR
2. Age <2 months with cough or difficulty breathing and at least one of the following: cyanosis, stridor at rest, hypoxia (O_2_ saturation <90%), head nodding, or general danger signs such as not drinking or breastfeeding, vomiting all intake, convulsions, lethargy or fainting, no movement or only when stimulated, OR
3. Age 2 to 59 months with cough or difficulty breathing and at least one of the following: tachypnea, chest in-drawing, cyanosis, head nodding, stridor at rest, hypoxia (O_2_ saturation <90%) or general danger signs such as not drinking or breastfeeding, vomiting all intake, convulsions, lethargy or fainting.

### Laboratory methods

Nasopharyngeal swab specimens were collected from each patient using a polyester swab that was placed in viral transport media, stored at 4-8 °C for ~ 24 h, then frozen at -20 °C and sent for laboratory analysis at *Centro de Estudios en Salud* (CES)-UVG where they were tested using real-time reverse transcriptase polymerase chain (rRT-PCR) reaction per standard CDC protocols for influenza A and B viruses. Specimens that were positive for influenza A were tested for subtypes using probes and primers developed by the CDC for H1 (i.e., seasonal H1), H3, and A(H1N1)pdm09 [29].

### Statistical analyses

We examined data from the VICo respiratory syndrome database for Santa Rosa department collected during June 2007 to September 2012, for Quetzaltenango department collected during February 2009 to September 2012, and for Guatemala City collected during November 2009 to December 2011. The primary outcome was laboratory-confirmed influenza infection among hospitalized patients enrolled in the study at one of these three sites. Data were analyzed using Statistical Analysis System 9.2 (SAS Institute, Inc., Cary, North Carolina, USA).

Patient characteristics – including demographic (age, sex, ethnicity, monthly income, size of household), clinical, and other risk factors variables – were tabulated by site and compared using chi-squared test (for categorical variables) and Wilcoxon-Mann-Whitney test (for continuous variables). Fisher’s exact test was used for categorical variables with fewer than five counts in any cell.

Unadjusted annual incidence rates of hospitalized influenza were calculated for Santa Rosa and Quetzaltenango using the projected population from Guatemala’s National Institute for Statistics (INE) for each department based on the 2002 census [30]. We adjusted for health facility utilization based on studies conducted prior to the implementation of the surveillance system by dividing the actual number of cases by an adjustment factor. In Santa Rosa, the adjustment factor was 0.33 for children < 5 years age, 0.75 for those five years and older [31]. In Quetzaltenango, the adjustment factor was 0.75 for children < 5 years and 0.5 for those five years and older [27, 32].

We extrapolated the populations based on past growth rates of 2.3% per year. We calculated age-specific incidence of patients hospitalized with influenza and estimated 95% confidence intervals (CI) using the Poisson distribution. Additionally, for 2008 in Santa Rosa, 2009 in Quetzaltenango, and 2012 for both, the denominator data were adjusted to reflect partial time at risk. We did not calculate the incidence for Guatemala City because of insufficient data to estimate healthcare utilization adjustment factors.

We conducted a cross-sectional analysis among all hospitalized patients to identify risk factors for severe disease, defined as being admitted to the intensive care unit (ICU) or in-hospital death. Logistic regression was used to estimate the odds of severe disease given a priori risk factors previously observed in other studies (age, sex, household size, ethnicity, end-point pneumonia, treatment received, sought care prior to hospitalization, and influenza type). Bivariate odds ratio were estimated with a 95% confidence interval. Those variables with statistically significant association at *p* < 0.05 level were entered into a model and a manual selection process was conducted until only variables at p < 0.05 remained in the final model. The final model estimated the adjusted odds ratio (aOR) of having severe disease and associated 95% confidence intervals (CI), adjusted for age and sex.

### Ethics

The *Vigilancia Integrada Comunitaria* (VICo) protocol was approved by human subjects committees of *Universidad del Valle de Guatemala* (UVG – local ethics approval) and Centers for Disease Control and Prevention (CDC), and accepted by *Ministerio de Salud Publica y Asistencia Social* (MSPAS). At screening, all patients presenting at the facilities were asked for verbal consent if ≥ 18 years or verbal assent if < 18 years old. At enrollment, written informed consent was obtained from patients ≥ 18 years old and from guardians of patients < 18 years old. In addition, patients between seven and 17 years old were asked for informed written assent.

## Results

Of 9866 patients who presented to a health facility with respiratory symptoms, 9610 (97%) verbally agreed to be screened, and 8847 (92%) enrolled into the VICo respiratory surveillance system, of which 6326 (72%) were enrolled and admitted at one of three hospitals. Among those enrolled at a hospital, 446 (7%) tested positive for influenza: influenza A was detected in 360 (81%), influenza B in 82 (18%), and influenza A and B in 4 (1%). Among 360 influenza A detections, 38 (11%) were type H1, 70 (19%) were type H3, and 197 (55%) were pandemic A(H1N1)pdm09 strain. Of note, 55 (15%) of the influenza A samples were not typed because of inadequate specimen volume.

**Fig. 1:**
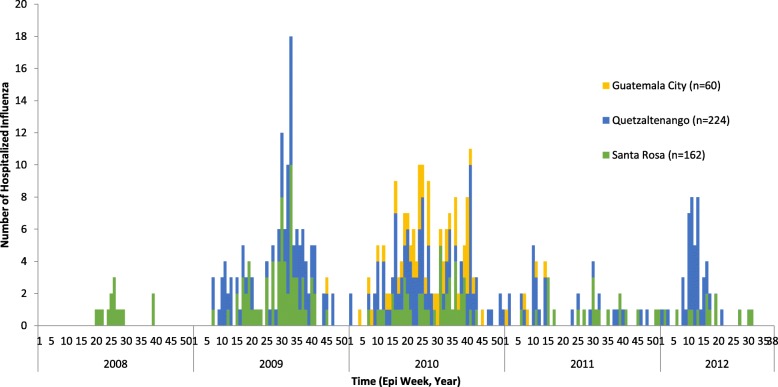
Hospitalized Influenza Patients in the VICo surveillance System by Type, Guatemala, 2008–2012

The number of influenza hospitalizations peaked at epidemiological week 33 of 2009 at 18 patients (Fig. 1). The effect of the A(H1N1)pdm09 epidemic was most evident in Santa Rosa with an increase in unadjusted incidence from 5.7 in 2008 to 27.5 per 100,000 in 2009. The number of hospitalizations attributed to influenza for 2008 (Santa Rosa Hospital only) was 14; for 2009 was 145 (Santa Rosa (*n* = 69) and Quetzaltenango hospitals (*n* = 76), and for 2010 was 184 (IGSS added). Over half the patients were male (*n* = 254, 57%) (Table 1).

**Table 1:** Demographic characteristics of hospitalized laboratory-confirmed influenza cases of the *Vigilancia Integrada Comunitaria* (VICo) Respiratory Surveillance System, 2008–2012

Characteristics	Guatemala City^a^*n* = 60	Quetzaltenango (Xela) *n* = 224	Santa Rosa (Cuilapa) *n* = 162	Total *n* = 446	*p*-value
Age (years)					
Median	1.2	5.9	2.1	2.4	< 0.01^b^
Mean	4.5	24.3	15.3	18.3	
Range	0.06–83.3	0.04–89.8	0.03–80.9	0.02–83.6	
Age Group					
0–2	38 (63)	86 (38)	81 (50)	205 (46)	< 0.01^c^
> 2–5	18 (30)	25 (11)	13 (8)	56 (13)	
> 5–15	0	13 (6)	21 (13)	34 (8)	
> 15–50	2 (3)	44 (20)	25 (15)	71 (16)	
> 50–65	1 (2)	26 (12)	9 (6)	36 (8)	
> 65	1 (2)	30 (13)	13 (8)	44 (10)	
Sex					
Male	30 (50)	132 (59)	92 (57)	254 (57)	0.46^c^
Ethnicity					
Indigenous	5 (8)	146 (69)	6 (4)	157 (36)	< 0.01^c^
Ladino	55 (92)	65 (31)	155 (96)	275 (64)	
Monthly Income					
≤ Q1,000^e^	5 (8)	109 (64)	121 (75)	235 (53)	< 0.01^c^
> Q1,000	54 (90)	62 (28)	40 (25)	156 (35)	
Missing	1 (2)	53 (24)	1 (0.6)	55 (12)	
Parent’s education^f^ for children under five (*n* = 261)					
None	5 (9)	23 (22)	26 (28)	54 (21)	< 0.01^c^
Primary	41 (73)	67 (63)	67 (72)	175 (69)	
Secondary	10 (18)	16 (15)	0 (0)	26 (10)	
Adult patient’s education (*n* = 185)					
None	1 (25)	24 (21)	20 (29)	45 (24)	0.03^d^
Primary	3 (75)	51 (45)	21 (31)	75 (41)	
Secondary	0 (0)	15 (13)	3 (4)	18 (10)	
Missing	0 (0)	23 (20)	24 (35)	47 (25)	

^a^ Instituto Guatemalteca Seguridad Social, Zona 9

^b^ Mann-Whitney-Wilcoxon test for equal median

^c^ Chi-square

^d^ Fisher’s exact test

^e^ Q = Quetzal with exchange rate during study of approximately 7.8 Q per dollar; Q1,000 = $128.21

^f^ Primary School is up through 6th grade (12 years of age), then Basic (12–15) and high school is up to 17 or 18

The majority of influenza hospitalizations, 285 (64%), had a history of febrile illness that started less than seven days prior to admission, but most patients (*n* = 217, 59%) did not have documented fever (≥38 °C) at admission. Other symptoms included cough (95%), abnormality during lung examination (85%), sneezing (77%), and sputum or phlegm (72%) (Table 2). Diagnosed pre-existing conditions were present in 101 (23%) of the patients, including chronic asthma (6%, median age = 41, heart disease (6%, median age = 15), and diabetes (4%, median age = 59).

**Table 2:** Clinical characteristics at admission of hospitalized patients with laboratory-confirmed influenza infection in the VICo Respiratory Syndrome Surveillance System, 2008–2012

Characteristics	Guatemala City *n* = 60	Quetzaltenango (Xela) *n* = 224	Santa Rosa (Cuilapa) *n* = 162	Total *n* = 446	Chi-square *p*-value
Fever started less than 7 days ago	28 (47)	153 (69)	104 (65)	285 (64)	0.01
Abnormality during lung examination	56 (93)	200 (90)	107 (73)	363 (85)	< 0.01
Tachypnea (fast breathing)	24 (40)	143 (64)	72 (44)	239 (54)	< 0.01
Diarrhea/Loose stool past 7 days	10 (17)	47 (21)	19 (12)	76 (17)	0.06
Cough	54 (90)	215 (96)	153 (96)	422 (95)	0.09
Sputum or phlegm	45 (75)	180 (81)	93 (58)	318 (72)	< 0.01
Sputum with blood	1 (2)	25 (11)	5 (3)	31 (7)	0.01
Any existing chronic condition	10 (17)	70 (31)	21 (14)	101 (23)	< 0.01
Heart Disease	4 (7)	21 (9)	1 (0.6)	26 (6)	< 0.01
X-ray results (*n* = 210)					
Alveolar consolidation/Pleural effusion	12 (34)	54 (54)	25 (31)	91 (43)	0.004
Other infiltrate/Abnormality	11 (31)	26 (26)	20 (25)	57 (27)	
None	10 (29)	19 (19)	33 (41)	62 (29)	
End-point Pneumonia (*n* = 239)	30 (71)	54 (50)	64 (72)	148 (62)	0.003
“Danger signs” in Children Under 5 (*n* = 261)					
Vomiting everything	12 (21)	38 (34)	20 (21)	70 (27)	0.22
Able to drink or breastfeed	24 (43)	58 (52)	37 (39)	119 (46)	0.06
Convulsions or attacks	5 (9)	10 (9)	13 (14)	28 (11)	0.03
Increased tendency to sleep	26 (46)	42 (38)	13 (14)	81 (31)	< 0.01
Immunization data					
Had vaccination card	40 (71)	63 (57)	40 (43)	143 (55)	0.004
Received influenza vaccine past 6 months (*n* = 261)					
Yes	0	3 (1)	1 (1)	4 (1)	
Missing	2 (3)	68 (30)	80 (49)	150 (3)	
Received pentavalent vaccine^a^ (*n* = 261)	40 (71)	82 (80)	56 (70)	178 (74)	0.62
Number of pentavalent doses^a^ (*n* = 261)					
1	5 (9)	16 (14)	8 (9)	29 (11)	0.30
2	6 (11)	11 (10)	12 (13)	29 (11)	
3	29 (52)	55 (50)	36 (14)	120 (46)	
Missing	16 (29)	28 (25)	38 (40)	82 (31)	
Received DPT (*n* = 261)					
Yes	2 (4)	0 (0)	5 (5)	7 (3)	0.04
Missing	41 (73)	91 (82)	62 (66)	194 (74)	

^a^ Pentavalent vaccine contains *Hemophilus influenza* B, Diphtheria, Pertussis (whole cell), Tetanus (DPT), and Hepatitis B are administered at 2, 4, and 6 months beginning in 2006. DPTis given at 18 months and 4 years

Of the 261 children aged < 5 years, IMCI general danger signs upon admission included inability to drink or breastfeed (51%) or vomiting (27%). Convulsions were report by 28 (11%) of the patients aged < 5 years. Fifty-five percent of 228 children aged < 5 years had a vaccination card available: residents of Guatemala City had the highest proportion (71%), followed by Quetzaltenango (57%) and Santa Rosa (43%). Four children (1%) had documentation of influenza vaccine in the previous six months. In contrast, 68% percent had received at least one dose of the pentavalent vaccine given at 2, 4, and 6 months containing *Hemophilus influenzae* B, diphtheria, whole cell pertussis, tetanus and hepatitis B, and 46% had received three doses (Table 2).

A higher proportion (22%) of persons smoking in the home was observed in Santa Rosa Department. The median size of the household was five persons (range 1–18). More than 66% of patients lived in a house with five or more people and 33% of households in Quetzaltenango had seven or more habitants. Seeking care elsewhere prior to hospitalization was reported by 57% of the patients or their care providers: 38% in Santa Rosa, 60% in Guatemala City, and 70% in Quetzaltenango (*p* < 0.01) (Table 3).

**Table 3:** Behavioral and environmental characteristics of hospitalized patients of the VICo Respiratory Surveillance System, 2008–2012

Characteristics	Guatemala City *n* = 60	Quetzaltenango (Xela) *n* = 224	Santa Rosa (Cuilapa) *n* = 162	Total *n* = 446	Chi-square *p*-value
Smoking by patient (*n* = 185)					
Yes	0 (0)	11 (10)	3 (4)	14 (8)	0.21
Missing	0 (0)	21 (19)	21 (31)	42 (23)	
Other people smoking in house					
Yes	8 (13)	26 (12)	35 (22)	69 (16)	0.04
Size of household					
1–4	25 (42)	75 (36)	48 (30)	148 (34)	0.01
5–6	24 (40)	66 (31)	82 (51)	172 (40)	
7+	11 (18)	70 (33)	31 (19)	112 (26)	
Sought care elsewhere before hospitalization					
Yes	36 (60)	148 (70)	57 (38)	241 (57)	< 0.01

The median length of hospitalization was five days (range = 0–77 days). Eighty-eight patients had severe disease (ICU admission or death). Eighty (19%) patients required intensive care including 30 (7%) patients who required mechanical ventilation. In-hospital deaths occurred in 28 (6%) patients. The proportion of hospitalized lab-confirmed influenza patients who experienced severe disease ranged from 5% in Guatemala City, 17% in Santa Rosa, and 24% in Quetzaltenango (*p* = 0.004).

Among 88 patients who were admitted to the ICU or who died, 67% were aged < 5 years of age (compared to 56% among those with less severe disease); 78% had diagnosed pre-existing conditions (compared to 75%); 63% of the 52 ICU patients who had a chest X-Ray had pneumonia (compared to 31%); 76% lived in a household with 5 or more persons (compared to 63%); and 72% had sought care elsewhere before being admitted to the hospital (compared to 53%). The median number of days from symptom onset to admission was 5 days (IQR = 6 days).

Children aged < 6 months of age comprised 85 (19%) of hospitalized patients attributed to influenza and 19 of 80 (24%) of patients who required ICU admission. Two (2%) of 85 children aged < 6 months died or 7% of the 28 deaths. Other decedents included 11 (6%) of 176 children aged 7–60 months, 6 (6%) of 105 person aged 5–50 years, and 9 (11%) of 80 patients aged > 50 years. Among all hospitalized patients, 26 (6%) were women of reproductive age (15–49 years old); two were admitted to the ICU, and one died.

Comparing patients admitted to the ICU or decedents with other patients, bivariate analyses suggest site, ethnicity, pneumonia, household size, and the seeking of care before hospitalization were associated with severe disease. Age and sex, did not seem to predict severe disease in this population (Table 4). Antiviral therapy information was available for only 268 patients, but among these, 97% received antiviral therapy. In multivariate analyses adjusted for age and sex, three characteristics remained independent risk factor for poor outcome: site, ethnicity, and pneumonia characterized by consolidation and/or large effusion. Compared with patients in Guatemala City IGSS hospital, patients in Quetzaltengo had five times and Santa Rosa had nine times the odds of experiencing a severe disease. The odds of having severe disease were four times (95% CI = 2–13) higher in patients who had indigenous ethnicity compared with Ladino patients. Those who had radiologically confirmed pneumonia had five times (95% CI = 3–11) the odds of experiencing severe disease compared to those who did not have pneumonia (Table 4).

**Table 4:** Risk factors for severe disease among hospitalized patients enrolled in VICo respiratory syndrome surveillance, 2008–2012 (*N* = 446)

Characteristics	Severe Disease^a^(*n* = 88)	Less Severe (*n* = 358)	Bivariate OR (95% CI)	Multivariate^b^OR (95%)
Age Group				
0–2	43 (21.0)	162 (79.0)	1	
2–5	16 (28.6)	40 (71.4)	1.5 (0.8–2.9)	
5–15	10 (29.4)	24 (70.6)	1.6 (0.7–3.5)	
15–50	8 (11.3)	63 (88.7)	0.5 (0.2–1.1)	
50–65	6 (16.7)	30 (83.3)	0.8 (0.3–1.9)	
65+	5 (11.4)	39 (88.6)	0.5 (0.2–1.3)	
Sex				
Male	48 (18.9)	206 (81.1)	1	
Female	40 (20.8)	152 (79.2)	1.1 (0.7–1.8)	
Site				
Guatemala City	3 (5.0)	57 (95.0)	1	1
Santa Rosa	32 (19.8)	130 (80.2)	4.7 (1.4–15.0)	9.5 (1.8–50.2)
Quetzaltenango	53 (23.7)	171 (76.3)	5.9 (1.8–19.6)	5.2 (0.9–29.7)
Ethnicity				
Latino	46 (16.7)	229 (83.3)	1	1
Indigenous	42 (26.8)	115 (73.2)	1.8 (1.1–2.9)	4.3 (1.5–12.5)
Existing Chronic Disease				
No	19 (18.8)	82 (81.2)	1	
Yes	67 (20.2)	264 (79.8)	0.9 (0.5–1.6)	
Pneumonia on Chest –X-ray (*n* = 239)				
No	19 (12.8)	129 (87.2)	1	1
Yes	33 (36.3)	58 (63.7)	3.9 (2.0–7.4)	5.2 (2.5–11.0)
Antiviral treatment received (*n* = 268)				
No	1 (16.7)	5 (83.3)	1	
Yes	50 (19.1)	212 (80.9)	1.2 (0.1–10.3)	
Type of flu				
A	74 (20.6)	286 (79.4)	1	
B	12 (14.6)	70 (85.4)	0.7 (0.3–1.3)	
A and B	2 (50.0)	2 (50.0)	3.9 (0.5–27.9)	
Household Size				
1–4	21 (14.2)	127 (85.8)	1	
5–6	35 (20.4)	137 (79.7)	1.5 (0.9–2.8)	
7+	32 (28.6)	80 (71.4)	2.4 (1.3–4.5)	
Sought care before hospitalization				
No	24 (13.3)	157 (86.7)	1	
Yes	62 (25.7)	179 (74.3)	2.3 (1.4–3.8)	

^a^ Defined as requiring intensive care unit (ICU) admission or death

^b^Adjusted for age, sex, and other variables in final model

**Fig. 2:**
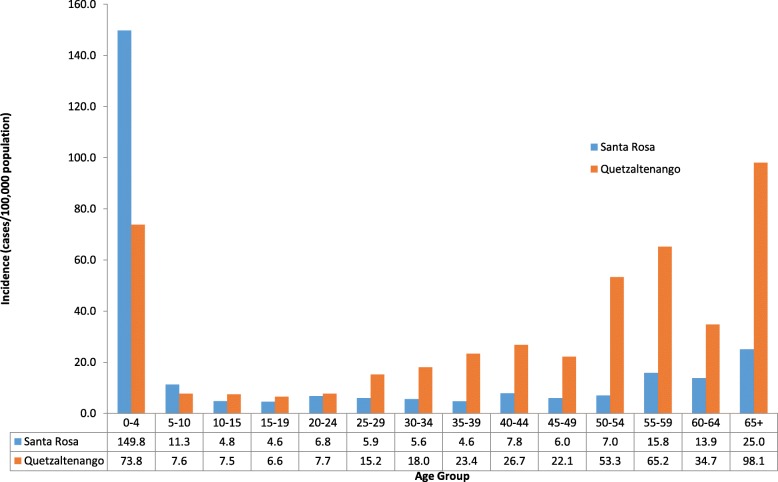
Adjusted Cumulative Incidence of Hospitalized Influenza in Guatemala, 2008–2012

Incidence of hospitalized influenza peaked in 2009 and 2010 during the A(H1N1)pdm09 pandemic. Figure 2 shows the health utilization survey (HUS) adjusted age-specific incidence of hospitalized influenza by site. Incidences were highest among children aged < 5 years and persons aged ≥65 years; children aged < 5 years constituted the greatest number of cases. The HUS-adjusted incidence was highest among children aged < 5 years in Santa Rosa in 2009 (291.8 cases/100,000 population), and highest among adults 65 years and older in Quetzaltenango in 2010 (129.1 cases/100,000 population) (Table 5).

**Table 5:** Adjusted age-specific incidence of hospitalized influenza in two departments in Guatemala, 2008–2012. Adjusted for healthcare utilization

		Santa Rosa			Quetzaltenango		
		Number of cases	Crude rate per 100,000 persons	Adjusted^a^ rate per 100,000 persons	Number of cases	Crude rate per 100,000 persons	Adjusted^a^ rate per 100,000 persons
2008	< 5 years	10	27.0 (13.0–49.7)	81.9 (54.7–115.8)	–	–	–
	≥ 65 years	0	0 (0–27.8)	0 (0–27.8)			
	All	14	5.7 (3.1–9.5)	14.5 (10.1–20.0)	–	–	–
2009	< 5 years	36	96.3 (67.4–133.3)	291.8 (239.4–351.7)	37	75.4 (53.1–104.0)	100.6 (73.9–132.1)
	≥ 65 years	3	22.1 (4.6–654.7)	29.5 (8.0–75.5)	9	60.9 (27.8–115.6)	121.8 (72.2–192.5)
	All	69	27.5 (21.4–34.8)	52.5 (43.8–62.1)	76	23.6 (18.6–29.6)	39.6 (32.9–46.9)
2010	< 5 years	30	79.4 (53.6–113.3)	240.6 (192.7–294.3)	44	79.2 (57.5–106.3)	105.5 (80.0–135.9)
	≥ 65 years	4	28.8 (7.9–73.8)	38.4 (11.7–84.0)	11	65.6 (32.2–115.5)	129.1 (80.9–195.5)
	All	46	18.0 (13.2–24.0)	43.8 (36.0–52.6)	84	22.8 (18.1–28.2)	37.6 (31.5–44.3)
2011	< 5 years	10	25.9 (12.4–47.7)	78.6 (52.5–111.0)	14	24.6 (13.4–41.3)	32.8 (19.4–51.0)
	≥ 65 years	4	28.2 (7.7–72.3)	37.6 (11.5–82.3)	4	22.9 (6.3–58.8)	45.9 (19.8–90.5)
	All	20	7.7 (4.7–11.8)	16.7 (12.1–22.4)	25	6.6 (4.3–9.8)	10.8 (7.7–14.6)
2012	< 5 years	8	30.3 (13.1–59.7)	91.8 (58.3–135.3)	16	41.0 (23.4–66.6)	54.6 (33.3–82.2)
	≥ 65 years	2	20.6 (2.5–74.5)	27.5 (4.3–82.6)	6	50.2 (18.4–109.3)	100.4 (51.9–175.5)
	All	13	7.3 (3.9–12.4)	17.3 (11.6–24.3)	39	15.1 (10.7–20.6)	26.0 (20.0–32.8)

^a^Adjusted for actual health seeking behavior of respondents in a health utilization survey if they were sick in the past 6 months

## Discussion

The VICo surveillance platform – which has dedicated nurses actively seeking patients with severe respiratory symptoms and PCR confirmation of influenza virus infection – demonstrates that influenza is an important cause of hospitalization and death in Guatemala. Children aged < 5 years and those aged > 65 have the highest incidence of hospitalization for influenza and the case fatality rate was highest in those 50 years and older (11%). In this study, we identified three characteristics of patients with severe disease or death: study site, ethnicity, and presumed bacterial pneumonia. Pre-existing heart, lung, and metabolic conditions were prominent in hospitalized patients. In addition, few patients presenting to all three hospitals seemed to have been vaccinated against influenza. Interventions to prevent infection and minimize serious outcomes should target these factors.

We estimated the cumulative incidence rate for hospitalized influenza at the two sites of the surveillance system. Overall, children < 5 years are at highest risk of hospitalization in both sites and for all years followed by those over 65 years. In general, the A(H1N1)pmd09 strain affected young persons more than those older than 50 years, a pattern seen world-wide. Younger age groups and older adults should be the targeted for vaccination [33]. A prospective study in Nicaragua documented high incidence of influenza, especially in infants 6–11 months, a vaccine eligible age [12]. In particular, better strategies to protect the youngest children (aged < 6 months) are needed as vaccination is not currently recommended for this age group. Immunization of pregnant women provides protection for women and their infants [34].

The present study clearly indicate children aged less than 5 years and persons 65 years and older have the highest incidence rates of influenza hospitalization. A strength of the present study is that cases were sought actively by study nurses and influenza virus was detected by sensitive PCR. Comparing incidence rates obtained in the present study with those estimated by attributing acute respiratory syndrome discharge diagnoses to the proportion of monthly influenza positive tests by indirect fluorescent antibody [9], crude rates in children aged less than five years are similar (Table 5). However, a major difference is observed in the incidence among persons aged 65 years and older. We observed incidence rates in 2009–2012 of 22–65/100,000 compared with 2–10/100,000 modeled using multipliers and influenza laboratory confirmation  through indirect immunofluorescence. The active surveillance in the present study highlights the importance of influenzaprevention in this older age group which can be underestimated through the multiplier method.

There are significant differences among the three study sites that might be important in prevention and treatment strategies. The median age of admission was highest in Quetzaltenango at 6 years of age compared with Santa Rosa at 2 years, and Guatemala City at 1 year. The difference might reflect health-seeking behaviors of guardians in the three sites, or might reflect differences in epidemiology, or different specialties at the hospitals. For example, the Cuilapa National Hospital in Santa Rosa is a regional referral center for neonates and children. The three sites have very different environmental, socio-economic and ethnic profiles that might require different approaches in prevention and managing influenza. For instance, at the Cuilapa hospital, patients reported lower income and educational levels, and often presented to the hospital as their first encounter with health care. As residents in the highlands of Quetzaltenango are often ethnically indigenous, education materials abou the prevention of influenza including vaccination information should be made available in the local Mayan dialect languages in addition to Spanish, the national language in Guatemala. Like in our study, indigenous persons, including those in the United States, can have higher rates of complications from influenza illness [35–41].

The clinical characteristics of hospitalized patients also provide some insights about prevention strategies for patients with pre-existing conditions. Among all hospitalized patients, 23% had a pre-existing condition consistent with studies elsewhere [7, 21]. Seventy eight percent of those who died had a history of a chronic disease. Patients with underlying heart, lung or metabolic disease should be a priority group for prevention messages and vaccination against influenza in Guatemala [18].

Review of personal immunization records revealed that only 1% of children aged < 5 years had documented influenza vaccine in the past 6 months. Patients aged < 5 years in Guatemala City had the highest proportion (71%) of vaccination card ownership, followed by Quetzaltenango (57%) and Santa Rosa (43%). Ropero-Alvarez et al reported that during the A(H1N1)pdm09 pandemic, vaccinations were procured between January and May 2010, but only 10% of the population was covered [42]. In addition, our findings suggest that some patients had not been fully vaccinated with the Penta vaccine, introduced in Guatemala in 2005, that provides protection against three respiratory pathogens: (*H. influenzae*, diphtheria and pertussis). Immunization cards may need to be updated to include specific locations for recording more recently recommended vaccines such as influenza and conjugated pneumococcal vaccine. Vaccination against *S. pneumoniae* with 13 valent conjugated vaccine was introduced in late 2012. This vaccine is expected to have an impact on one of the major determinates of severe disease, pneumonia. Furthermore, utilization of the electronic vaccine registry in SIGSA Web may be useful in documenting individual vaccination and overall coverage [43]. Finally, health surveys in Guatemala should routinely include vaccine coverage and acceptability among health providers and target groups (ENSMI study).

Compared to patients in Guatemala City, those in Santa Rosa and Quetzaltenango had higher odds of experiencing poor outcomes, although the association was statistically significant only for Santa Rosa. In addition, indigenous patients also had higher odds of dying or needing ICU compared with Ladino patients. These two factors combined suggest the possibility of a health inequity situation among the sites, and between these two major ethnic groups.

Geography and environment may play an important factor as well since the city of Quetzaltenango is located at 7640 ft resulting in much lower mean temperatures (14.7 °C; 58.5 °F) compared with Cuilapa at 2913 ft (889 M) with a mean temperature of 24.6 °C (76 °F). Quetzaltenango and the surrounding highlands have many nights each year below freezing temperatures; for example, the average low in January is 2.3 °C (36.1 °F). Likewise, the common practice of cooking on open fires inside buildings and use of open fires for warmth among Mayans might increase the risk of respiratory infections [44]. The increased odds of severe disease among indigenous people in the rural highlands might be due to less access to the appropriate care, delayed treatment, nutritional, cultural, environmental, or other factors.

The IGSS Zona 9 hospital in Guatemala City, financed through workers and employer’s contributions, may have better-equipped health care facilities that are more easily accessed compared with the other two sites. The vaccine procurement system is also different from the public sector. Even so, none of the 60 cases had documented receipt of influenza vaccine in the six months prior to admission.

This study has several limitations. First, we did not collect detailed information about outpatient illness and/or care received at health posts and centers prior to hospitalization; therefore, we were not able to investigate the risk factors for hospitalization itself. Second, the data are further limited by the denominator population estimates from INE, and we lacked a population data estimate incidence for Guatemala City. Although the healthcare utilization data provided additional precision to the incidence estimate, these were conducted in Santa Rosa in 2006 and Quetzaltenango in the first half of 2009, before the major number of cases of the 2009 influenza A(H1N1)pdm09 pandemic occurred. Therefore, the reported utilization might have imprecisely estimated the actual use of facilities from 2009 through the present, after the pandemic. Although our case definition was purposely sensitive  and data collectors were trained on them, it is possible that some cases might be missed, resulting in an underestimation of the incidence. Finally, our data about influenza illnesses in pregnant women was incomplete. Data was not collected on current pregnancy status on all women early in the study. Nonetheless, pregnant women are a high risk group for severe complications and therefore vaccination during pregnancy is warranted [18].

## Conclusions

This study demonstrates high rates of influenza-associated morbidity and mortality in Guatemala. Consistent with current global recommendations, this study highlights the potential value of vaccinating high risk groups, such as pregnant women, children aged > 6 months, persons aged > 60 years, those with pre-existing health conditions, and health care workers [18, 45, 46]. Our data suggest the value of exploring the impact and cost effectiveness of influenza vaccination among SAGE target groups including ethnic minorities in Guatemala. Utilization of quadrivalent vaccines, high dose or adjuvant vaccines might also be useful in tropical middle income settings like Guatemala [17], but the main emphasis should be on increasing vaccine coverage [47]. Vaccine coverage surveys need to be completed periodically to monitor and improve influenza immunization rates [48]. Furthermore, influenza vaccinations can prevent illnesses that may lead to unwarranted antibiotic use and bacterial resistance among persons with viral infections. Because we identified pneumonia on chest X-ray as an independent risk factor for severe illness, the effects of introduction of conjugated pneumococcal vaccine in Guatemala in 2012 against *S. pneumoniae* – the major cause of bacterial superinfection of influenza – should be monitored [49]. Specific treatment of influenza with neuraminidase inhibitors early in severe illness should become standard [50, 51]. Future health education and vaccination efforts should be tailored to fit the different epidemiologic, ethnic, and geographic profiles found across the country.

## Abbreviations

aOR: Adjusted odds ratio
ARI: Acute respiratory infection
BMI: Body mass index
CDC: Centers for Disease Control and Prevention
CES: *Centro de Estudios en Salud*
CI: Confidence interval
HUS: Health utilization survey
ICU: Intensive care unit
IGSS: *Instituto Guatemalteco Seguridad Social*
IMCI: Integrated Management of Childhood Illness
INE: National Institute for Statistics
IQR: Interquartile range
MSPAS: *Ministerio de Salud Publico y Asistencia Social*
PAHO: Pan American Health Organization
PDA: Personal digital assistant
rRT-PCR: Real-time reverse transcriptase polymerase chain reaction
SAS: Statistical Analysis System
TAG: Technical Advisory Group
UVG: *Universidad del Valle de Guatemala*
VICo: *Vigilancia Integrada Comunitaria*
WHO: World Health Organization
